# Bioengineering the Bone Marrow Vascular Niche

**DOI:** 10.3389/fcell.2021.645496

**Published:** 2021-04-28

**Authors:** Thomas Bessy, Tomer Itkin, Diana Passaro

**Affiliations:** ^1^Leukemia and Niche Dynamics Laboratory, Université de Paris, Institut Cochin, Institut National de la Santé et de la Recherche Médicale, Centre National de la Recherche Scientifique, Paris, France; ^2^Division of Regenerative Medicine, Ansary Stem Cell Institute, Department of Medicine, Weill Cornell Medicine, New York, NY, United States

**Keywords:** bone marrow, vascular niche, endothelial cells, bioengineering, bioprinting, manufacturing, organoid, microfluidics

## Abstract

The bone marrow (BM) tissue is the main physiological site for adult hematopoiesis. In recent years, the cellular and matrix components composing the BM have been defined with unprecedent resolution, both at the molecular and structural levels. With the expansion of this knowledge, the possibility of reproducing a BM-like structure, to ectopically support and study hematopoiesis, becomes a reality. A number of experimental systems have been implemented and have displayed the feasibility of bioengineering BM tissues, supported by cells of mesenchymal origin. Despite being known as an abundant component of the BM, the vasculature has been largely disregarded for its role in regulating tissue formation, organization and determination. Recent reports have highlighted the crucial role for vascular endothelial cells in shaping tissue development and supporting steady state, emergency and malignant hematopoiesis, both pre- and postnatally. Herein, we review the field of BM-tissue bioengineering with a particular focus on vascular system implementation and integration, starting from describing a variety of applicable *in vitro* models, ending up with *in vivo* preclinical models. Additionally, we highlight the challenges of the field and discuss the clinical perspectives in terms of adoptive transfer of vascularized BM-niche grafts in patients to support recovering hematopoiesis.

## Introduction

Recent advances in bioengineering have dramatically improved the ability of tissue reconstitution with partially restored functions, for both fundamental research and regenerative medicine. In the specific case of the bone marrow (BM), the reconstitution of a functional multicellular unit is of therapeutic interest both for BM grafts and for bone repair (Oryan et al., 2014; Kawecki et al., 2018). Besides the generation of organ sized tissue substitutes, generating a BM-on-a-chip also have multiple interests. Indeed, there is a need of *ex vivo* cultures for stem cell clinical expansion, but it is also a necessity to have strong physiological-like models for fundamental research, that for ethical reasons might replace in the future the necessity for *in vivo* studies, also providing physiologically relevant human model systems. However, as of today the human hematopoietic niche complexity is still not fully understood, and despite the recent advances in murine studies, the function and cellular organization/composition in humans is vastly missing. As human genetic *in vivo* manipulation studies are not feasible experimentally and ethically, tissue bioengineering and modeling represents a straightforward approach to progress our understanding of human hematopoiesis. Mimicking tissue function “on a chip” can help to grasp which are the minimal components necessary for proper hematopoietic niche homeostasis or for instigation and propagation of a malignant phenotype. “Niche-chips” can complement drug tests, which are not always translatable with 1:1 accuracy to human from rodent models, to assess a specific compound impact on multiple types of human cells and to decipher the mechanisms of action. “Niche chips” can be used for the comprehension of radio-chemoresistance in investigation of cancer behavior without inducing diseased animal models, which represents an ethical step forward. All in all, the increase in bioengineered chips in biology reflects an overall need and scientific will to move forward to superior physiological and ethical models relevant for the design of new clinical therapies.

### State of the Art

Considering the therapeutic interest of hematopoietic stem cells (HSCs), which were the first type of stem cells used in modern medicine, it is understandable that there is an unmet need to obtain the capacity to robustly expand these cells *ex vivo* while maintaining stemness properties. Early enough, it became very clear that controlling balanced HSC proliferation and differentiation *in vitro* is a challenging task to achieve outside of the physiological BM microenvironment. For this reason, the vast majority of BM mimicking studies focus on HSC stemness maintenance. “True” HSC maintenance and long-term repopulation capacity have been the major readouts for the quality of the engineered BM-like tissues. These studies aim at reproducing a minimalistic HSC-maintaining or HSC-“activating” niche (Ingavle et al., 2019).

The first major step in the field has been using a layer of feeder cells (Blackburn and Patt, 1977), particularly mesenchymal stromal cells (MSCs), to sustain HSC potential in co-culture conditions. This discovery has also been the first proof of concept of the importance of the cellular environment for HSC regulation. Therefore, the role of MSCs has been the main focus of further studies compared to any other niche cell types (e.g., hematopoietic, endothelial, nerve cells), despite the numerous studies showing the role of other niche components in HSC regulation *in vivo* (Pinho and Frenette, 2019).

Notwithstanding this progress, simple HSC-MSC co-cultures rapidly found their limitations, as HSCs slowly but inevitably exhausted and lost their stemness potential. To enhance the co-culture system, many physiological parameters and paracrine niche derived factors have been suggested to be key for the reproduction of a better mimicking environment for stem cells. Notably, the oversimplified 2-dimensional aspect of these co-cultures has been pointed out as a major flaw.

**FIGURE 1:**
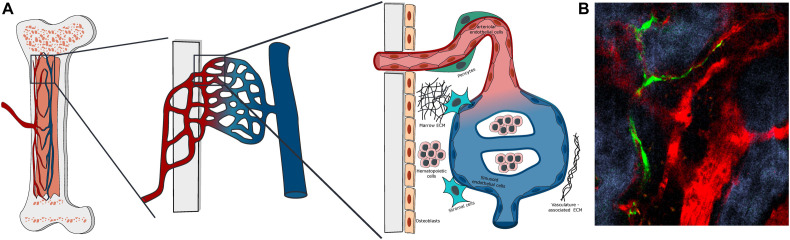
Bone marrow vascular structure. **(A)** Scheme of the organization of BM vasculature at different magnifications. The BM vasculature is highly interconnected with the tissue matrix, with small arterioles covered by pericytes entering the marrow from the bone and merging with the venous system via a network of sinusoidal capillaries. The vascularized marrow space is populated by hematopoietic and stromal cells embedded in a unique extracellular matrix. **(B)** Representative Z-stack two-photon microscopy image of vascular organization in the mouse calvarial bone marrow, with Nestin-GFP peri-arteriolar pericytes (green), TRITC-dextran labeled vascular lumen (red) and second harmonic generation (SHG) bone matrix (blue).

Indeed, it has been shown that HSCs benefit from multiple signals coming from the complex 3D microenvironment such as the enhanced exposure to ECM-derived factors, the physical properties of the microenvironment (e.g., rigidity, oxygen tension), and the heterogeneity of contacting cells, composing the multicellular niche unit (Figure 1). A simple increase in dimensionality by using MSCs organized in spheroids has proven of moderate interest (Isern et al., 2013; Schmal et al., 2016; Futrega et al., 2017; Lewis et al., 2017), showing that the plurality of signals (physical and/or biological) are crucial when reproducing a semi-marrow environment. Thus, the choice of materials is determinant in the design of an engineered BM chip (Nichols et al., 2009; Raic et al., 2014; Severn et al., 2019). For instance, ECM proteins impact HSCs in several ways such as direct biological HSC regulation (Wilkinson et al., 2019), by their tethering, and by generation of mechanical forces (Trappmann et al., 2012). Additionally, ECM proteins have the ability to immobilize biological factors (Mahadik et al., 2015) differently interacting with stem cell in comparison to their soluble state. ECM substrate elasticity by itself can also play a role in HSC regulation (Holst et al., 2010; Choi and Harley, 2017; Gvaramia et al., 2017). Furthermore, once HSCs are included in a 3-dimensional material, the pore-size as well as the cell density in these pores will become a novel important parameter impacting on the concentration of autocrine signaling, flow rate, and shear stress (Müller et al., 2015). A three-dimensional material will also bring a different oxygen tension compared to 2D culture (Sharma et al., 2012), to which HSCs are sensitive (Jing et al., 2012). Altogether, a profound scaling-up in dimension and complexity is required to achieve a high-quality BM-like mimicking system.

As 3D engineered BM tissues are only recent and still under development, only few studies have focused on the importance of upbringing a vascular system into the tissue-like chip format. The role of vascularization has already been shown as essential to support organ function and regulation of cell fate choice decisions, organization and differentiation (Itkin et al., 2016). Up until recently, the vascular system has been widely depreciated in the field of BM bioengineering to support HSCs, as can be demonstrated by the small number of studies using endothelial cells (ECs) in co-culture (Khan et al., 2014). This is in spite of the fact that the importance of the vascular system in the BM has been largely demonstrated by *in vivo* animal studies (Ohneda et al., 1998; Acar et al., 2015; Itkin et al., 2016; Xu et al., 2018). First, the vascular system brings a structural organization within the BM microenvironment, defining specific angiocrine, nutrient, oxygen and metabolite gradients. Furthermore, it provides distinct types of specialized niches participating in the heterogeneous multifactorial signaling supporting HSC as well as other hematopoietic and stromal cell types (e.g., MSCs, osteolineage cells) (Langen et al., 2017; Chen et al., 2019). As in other organs, BM vasculature forms a restrictive barrier between the marrow environment and the rest of the blood flow, and it is involved in cellular exchanges, regulating homing, mobilization and tumor metastasis (Itkin et al., 2016; Carlson et al., 2019). Recent advances in whole tissue imaging and deep learning (Saçma et al., 2019) in mouse models have allowed to study the BM vascular network with unprecedent resolution, and specific endothelial cells lining specialized vessels have been defined as central actors of bone remodeling and cellular exchanges in homeostasis (reviewed in Hendriks and Ramasamy, 2020). Moreover, analytical integration of recently published data sets for BM stroma ‘omics analysis, on the single cell levels, allows the identification of additional stromal sub-types, revealing an undefined transitional population of ECs to be considered (Dolgalev and Tikhonova, 2021). The BM vascular niche has also an important function in hematologic as well as solid cancer development and progression (Batsivari et al., 2020; Haider et al., 2020). ECs in the BM have been shown as a fundamental source of angiocrine factors that promote development and survival of T-cell acute lymphoblastic leukemia (ALL) in the BM niche (Passaro et al., 2015; Pitt et al., 2015). Moreover, the vascular niche functionality is highly disrupted by the presence of acute myeloid leukemia (AML), a process that seems to involve specific types of vessels and cause an architectural abnormality and overall dysfunction of perfusion and permeability of the vascular tree. Moreover, this vascular disruption is retained and thus may facilitate leukemic relapse (Passaro et al., 2017b; Duarte et al., 2018). In some types of solid tumors, the endosteal niche is the primary site for metastasis, and the role of specialized endosteal vessels in this process has yet to be fully understood (Haider et al., 2020).

Overall, the vascular system is a pivotal actor in the physiological BM environment, whose role regarding hematopoietic regulation, bone activity and cancer development cannot be ignored in BM mimetic systems. The technical complexity of generating a structured vascular network explains why it has been neglected, comparing to other BM components.

### Challenges

**FIGURE 2:**
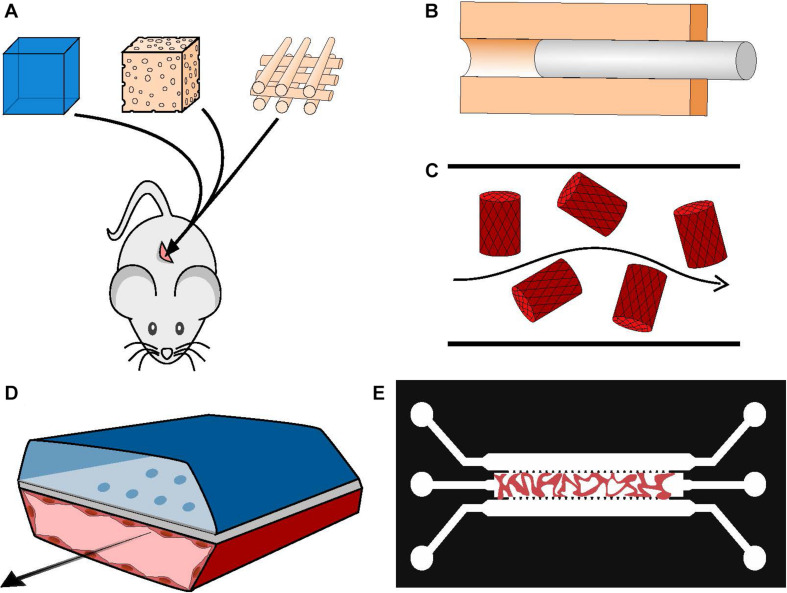
Manufacturing vascularized BM systems. **(A)** Implantation of mesenchymal and endothelial cell-loaded hydrogels, sponges or printed scaffolds subcutaneously in immunodeficient mice. The resulting vasculature will be anastomosed to mouse host vasculature. **(B)** Generation of a hollow tube in a 3D matrix through the use of sacrificial material; the channel walls can be seeded with ECs afterward. **(C)** Cell-loaded hydrogel islets are seeded with a layer of ECs and placed in a microfluidic channel with a continuous flow, forming an inverted vascularized system (Khan et al., 2012). **(D)** Formation of organ-on-chip systems with two parallel hollow tubes separated by a porous membrane, one containing tissue specific cells and the other coated with ECs and perfused with medium to serve as a functional vascular system (Huh et al., 2013). **(E)** Generation of self-assembled and perfusable vascular network in a microfluidic chip (see dedicated Box 1).

Any successful *ex vivo* engineered chip aiming to mimic the BM microenvironment will have to replicate a similar 3D architectural structure that will allow HSCs to freely interact with their neighboring niche cells without any physical constrains, in the presence of semi-physiological biophysical forces such as fluidic flow and application of shear stress on vascular beds. The composition of the ECM should also be considered and well defined as the final goal is to have the stem cells to reside in a physiological-like BM environment, with a similar range of stiffness/rigidity, that can be also translational and applicable for further clinical use (unlike the classical matrigel applied today for most *ex vivo* organoid assays). Thus, the main challenge in engineering a fully functional vascularized BM is reproducing both the cellular heterogeneity and the compartmentalized organization found *in vivo*. For this purpose, the high complexity of the system requires the identification of key elements in order to reproduce a minimalistic functional version of the tissue (Figure 2). To achieve those goals there are several strategies depending on what is the readout used for a functional niche such as: normal and leukemic HSC maintenance and development, normal lineage development, or cancer metastasis studies. Two main technical challenges can be foreseen in the production of such an engineered tissue. First, the presence of a unique and heterogenous network of vasculature with a specialized organization and cellular composition with the requirement of the ability to deposit and/or to structure distinctly defined cell types in the right position, with a high precision. Secondly, the BM has one particular challenging property to engineer: the presence of a dual environment, divided to the central marrow and the endosteal regions, that have very distinct physical properties and ECM composition (Zhang et al., 2019). The ECM rigidity at different scales dictates the use of different fabrication methods. Also, bone components are naturally opaque, making them non-friendly materials for observation by microscopy. Here, we will overview the attempts of the scientific community to achieve a vascularized BM mimicking tissue and discuss what are the next avenues in the field at the cross-road between cell biology and tissue bioengineering.

## Generation of Models for Experimental Approaches

### In vitro

#### First Attempts of Vasculature

**FIGURE 3:**
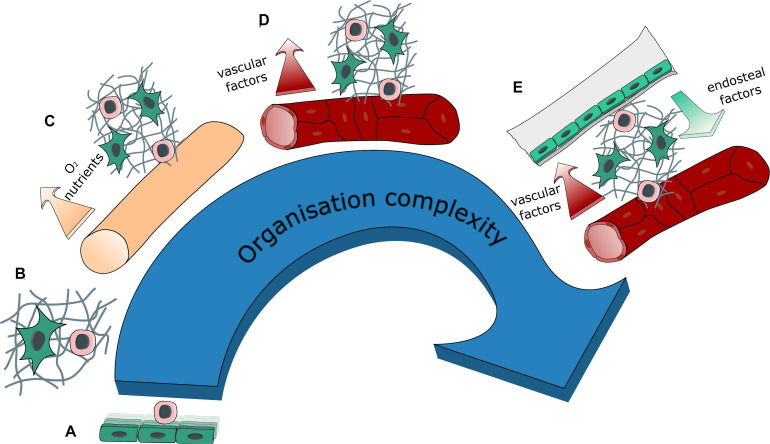
Evolution of system complexity over time. Evolution in the organization complexity of engineered BM models with: **(A)** 2D co-cultures (feeder cells), **(B)** 3D co-culture within a hydrogel, **(C)** introduction of a source for medium renewal with either perfusion or the creation of a hollow tube, thus generating compartments within the tissue, **(D)** introduction of vasculature (self-assembled or engineered) bringing contact-dependent interaction in addition to compartmentalization, **(E)** introduction of a bone environment generating a segmented endosteal and vascular regions, with different ECM and cellular contents as well as different physical properties.

Beside the ethical aspects, system simplicity, and the cost-advantage compared to animal models, one of the main advantages of *in vitro* BM mimetic semi-physiological systems is the possibility to construct a fully humanized modular system in which cellular components can be added or removed. This modular system can have different layers of complexity (Figure 3) and can be used for *ex vivo* manipulation and expansion of human hematopoietic stem and progenitor cells, as well as for studying lineage developmental process, and for screening drug or therapeutic treatment efficiency, along with tumor resistance properties.

In an attempt to support HSC function *in vitro*, many groups are using co-culture systems with MSCs in casted or 3D printed hydrogels/scaffolds (Leisten et al., 2012; Sharma et al., 2012; Ventura Ferreira et al., 2012; Raic et al., 2014; Zhou et al., 2020). If any form of vascularization is absent from this kind of model, it has been noticed that MSCs express a number of pro-angiogenic factors and in particular VEGF (Sharma et al., 2012). In general, MSCs and specifically BM-derived MSCs (BM-MSCs) support vascularization in presence of ECs (Au et al., 2008), and the two components (mesenchymal and endothelial) may provide a mutual niche and support each other via several factors essential for the homeostasis of the BM (Raida et al., 2006; Hu and Olsen, 2016). It has also been reported that BM-MSCs can support the vascular system by participating in the architectural blood vessel structure formation and maintenance, functioning as vessel lining pericytes sheathing the tubular structures (Chamberlain et al., 2012). All in all, this shows that the currently used models already have the baseline that is able to support the establishment of an organized vasculature *ex vivo*.

Without trying to reproduce a complex vascular structure, some groups introduced ECs in their *in vitro* systems to enhance HSC maintenance and to preserve functional stemness (Table 1). More than 25 years ago it was already demonstrated that human ECs support adhesion and development of human hematopoietic progenitor cells in *ex vivo* co-culture conditions (Rafii et al., 1994, 1995). However, prolonged culturing of ECs *in vitro* requires the presence of serum in the culturing media, which abrogates proper stem cell function and maintenance, and thus results in a failure to expand HSC on top of supportive endothelial niche cells (Radtke et al., 2019). A breakthrough in the field was achieved when it was discovered that the adenovirus derived E4orf1 peptide gene product can selectively enhance survival of vascular-niche forming ECs in prolonged serum free culture conditions via phosphorylation and activation of AKT (Zhang et al., 2004; Seandel et al., 2008). Later, this E4orf1 EC platform, as well as ECs transduced with constitutively active form of myrAKT, were shown to support *ex vivo* maintenance and enhanced self-renewal of both mouse and human HSCs (Butler et al., 2010, 2012; Kobayashi et al., 2010). Moreover, E4orf1 vascular niche cell platform was used to interrogate mechanisms of malignant hematopoietic lymphoma and leukemic cell expansion, development, resistance to myeloablative insult, and it even supported reprogramming-induced conversion of adult EC into HSPCs (Cao et al., 2014; Poulos et al., 2014; Sandler et al., 2014). Thus, E4orf1 ECs, with enhanced survival capacity, serving as vascular niche cells, allow the generation of *ex vivo* system model, to study and expand various types of hematopoietic cells interacting with ECs in a simplistic 2D semi-physiological microenvironment.

**TABLE 1:** Human endothelial cell sources.

Endothelial cell source	Tissue	Supporting stroma cells	Application	References
HUVEC	Umbilical cord vein	_ BM-MSC Multiple My eloma stromal cells Osteo differentiated BM-MSC HS5, HS27a, BM-MSC BM-MSC, Osteo differentiated BM-MSC hFOB, NHLF BM-MSC, hFOB Rabbit BM-MSC	*in vitro*	Jusoh et al. (2015) Khan et al. (2012), Jeon et al. (2014), Chou et al. (2020), Bray et al. (2017), Marturano-Kruik et al. (2018), Cui et al. (2016) de la Puente et al. (2015) Bersini et al. (2014), Jeon et al. (2015) Kotha et al. (2018) Nelson et al. (2019), Chiesa et al. (2020) Bessy et al. (2020) Ma et al. (2020) Zhang et al. (2017)
		BM-MSC	*in vivo*	Baldwin et al. (2017), Cidonio et al. (2020)

E4ORF1	Umbilical cord vein (E4ORF1 gene)	BM-MSC	*in vivo*	Abarrategi et al. (2017), Passaro et al. (2017a)
			*in patients*	Scordo et al. (2020)

R-VEC	Umbilical cord vein Dermal microvasculature Cardiac microvasculature + ETV2 gene Adipose tissue derived ECs Aortic derived ECs Pulmonary microvasculature	_ mouse pericytes	*in vitro in vivo*	Palikuqi et al. (2020)

AT-EC	Adipose tissue	AT-MSC	*in vivo*	Ghanaati et al. (2010)

EPC	Cord blood	_ BM-MSC, MS-5, adipose and osteo differentiated BM-MSCs hOB, BM-MSC	*in vitro*	Di Buduo et al. (2015) Braham et al. (2019) Glaser et al. (2020)
	Peripheral blood	BM-MSC	*in vivo*	Chen et al. (2012)

BM-EC	Sinusoidal and arteriolar BM endothelial cells	BM-MSC, osteo-differentiated BM-MSC	*in vitro*	Aleman et al. (2019)
	Bone marrow	BM-MSC	*in vitro*	Schaeffer et al. (2020)

HMVEC	Dermal Microvasculature	BM-MSC	*in vitro*	Mejía-Cruz et al. (2019)

In attempt to add complexity to mimic the BM microenvironment, Braham et al. (2019) used endothelial progenitor cells isolated from cord blood and allowed them to form a complex cellular environment together with MSC-derived adipocytes and osteoblasts. These culture conditions can be potentially used to maintain HSCs over long periods without the addition of any cytokines, as the mesenchymal and endothelial compartments should provide all the required cytokine and angiocrine factors. Additionally, this system can be used to study basic interactions like intercellular contact and cellular organization. It has been noticed, in these described systems, that the clustering of ECs and HSCs resembles a sinusoidal like formation. Similarly, HSCs were also cultured in a levitating cellular sphere, in association with BM-MSCs and ECs as a mean to recreate a multicellular niche (Mejía-Cruz et al., 2019). However, the investigation of cell organization is limited in all these models by the absence of cell compartmentalization and the dearth of a proper 3D vessel structure formation.

BM aspirates have also been used to recapitulate a multicellular environment for cancer cells. de la Puente et al. (2015) showed that multiple myeloma cells are supported by the combined presence of stromal and endothelial cells. The multi-culture in ECM hydrogels may reproduce a hypoxia gradient as well as a drug concentration gradient. Hypoxic gradient may establish the first step in compartmentalization, as in the BM where distinct compartments exhibit differential oxygenation statuses (Spencer et al., 2014), which can be altered in tumorigenic niches or following myeloablative treatment. Thus, the upbringing of compartmentalization can be achieved by the limited diffusion capacity within the gel forming the basis for the *in vitro* co-culture. This limitation also brings a nutrient shortage above a certain dimension, leading to cell death, similarly to pre-vascularized solid tumors. This shortage of nutrients can be another motivation for the generation of a functional perfusable vasculature that will allow proper nutrient and oxygen distribution in culture conditions.

To overcome this issue of nutrient shortage, several groups have applied a continuous slow flow rate to their hydrogels in a perfusion chamber (Wuchter et al., 2016; Bourgine et al., 2017, 2018; Rödling et al., 2017, 2018; Sieber et al., 2018). This allowed the routing of nutrients toward cells as well as some kind of interstitial fluid flow generating mechanosignals. Flow induced shear stress can activate cells through mechanotransduction pathways, and several groups have demonstrated that perfusion, flow, and shear rate stimulate the production of pro-angiogenic factors such as VEGF and Ang1 in MSCs and Osteoblasts (Bourgine et al., 2017).

#### Introduction of Organization

The simplest level of vascular organization that can be brought is by introducing BM cultures to hydrogels in a chip with compartmentalized areas: this kind of chip should have a dedicated reservoir for both nutrient-containing medium and for marrow cells (stromal and hematopoietic). This anisotropy in the layout would also create a non-homogeneous nutrient distribution and thus a certain level of compartmentalization. Ideally these compartments would be separated by a membrane or by a layer of ECs to form a regulatory barrier for chemical exchanges and to define specific multi-cellular regions. This kind of artificial separation was first experimented by Khan et al. (2012) who generated small MSC-containing gel beads, which were further coated with ECs and then introduced into a microfluidic channel where medium flow can be applied. Although the authors did not aim to mimic a physiological BM environment, they did demonstrate that this cellular organization combined with flow presence, favors MSC differentiation toward pericytic smooth muscle cells (SMCs) and instructs their alignment around ECs. However, the authors did mention that continuous non-laminar flow could negatively impact ECs over prolonged time periods.

Aleman et al. (2019) applied a technique of inverted vasculature (in which the medium is surrounding the gel structure) by flowing medium over an hydrogel containing ECs and MSCs in a microfluidic chip, like an underwater dome structure. This method, despite having no barrier separating the two compartments, could be used as a model for studying cellular exchanges between the BM and the bloodstream.

The next design presents the formation of a linear tube, separated from the cellularized matrix by a porous membrane of defined permeability (Allenby et al., 2018, 2019). This tube/membrane formation mimics a blood vessel barrier: a source of nutrients and incoming cells, but it also serves as evacuation path for secreted factors and extravasating cells. The matrix was constructed as a collagen-coated polyurethane scaffold with an average pore size of 180 μm and it is separated from the feeding channel by a membrane with an average pore size of 0.2 μm. The matrix was then seeded with cord blood-derived mononuclear cells (CB-MNCs) and cultured for several weeks. This culture system allowed self-organization and developmental specialization of cells in culture, with the majority of the cells aggregating around the pseudo-vessel structure toward the nutrient and oxygen source. Among the stromal cells observed in the chip, the authors noted Stro-1^+^ mesenchymal cells, OSX^+^ osteoprogenitors, OPN^+^ osteoblasts and in addition also elongated CD34^+^ endothelial precursor cells. Interestingly, mature osteoblasts were found distal to the vessel, in a more hypoxic region, therefore demonstrating the self-organization of a second compartment in the culture. In this multicellular chip, hematopoietic cells clustered around specific supporting cells, while macrophages where found in the middle of the tissue matrix and erythroid precursors clustered with OSX^+^ cells distally to the central vessel. This heterotypic cell interaction supported both erythropoiesis and the extravasation of mature red blood cells.

#### EC Lined Channels

In the above described model, an artificial membrane substituted a structured layer of ECs, which act as a dynamic barrier with active pumps and different regions of given perfusion/permeability. This membrane could not reproduce the specialized *in vivo* function of BM vessels. Tubular structures lined with ECs allow the introduction of a cellular barrier, and a novel degree of organization authorizing interaction between ECs and neighboring or migrating cells.

Chips for organ engineering developed by Ingber’s group, is an example for such an approach. In this system model, there are two channels separated by a porous membrane, the first one with the recapitulated tissue, and the second one coated with a layer of ECs to mimic the vasculature. This kind of chip has been used to engineer multiple types of organs (Bhise et al., 2014). It has been used for the first time in BM engineering by Bersini et al. (2014), with a central channel containing BM-MSC derived into osteoblast in a collagen gel and a lateral channel covered by a layer of ECs. In this particular study, this BM on chip system was used to model breast cancer cell invasion and bone metastasis. This chip, besides being a model for drug testing, recapitulated a minimal version of the marrow barrier and environment and allowed the observation of cancer cell interaction with specific BM cellular components.

Parenthetically, a similar construction was also used with a particular focus on hematopoiesis. Chou et al. (2020) used a chip with a fibrin/collagen matrix containing human CD34^+^ HSPCs and human BM-MSCs, it was kept in culture for several weeks. An EC-lined channel was feeding the main matrix through a porous PDMS membrane. This chip allowed the recapitulation of extended myelo-erythroid proliferation and differentiation. The hematotoxicity generated by chemical treatment (5-FU, and other novel drug candidates) or by radiotherapy was investigated using this chip. The effect was more potent compared to standard and static 3D cultures, demonstrating once again the relevance of engineered chips. Finally, the chip also demonstrated its ability to model the genetically defective hematopoiesis of patients with Schwachman–Diamond syndrome. However, no particular focus was made on the overall cellular organization among hematopoietic and stromal cells. Of note, no specific organization was observed along the layer of ECs since these lined-up ECs were separated from HSPCs and BM-MSCs by a porous membrane.

Endothelial/hematopoietic cell interaction was also observed in chips built for platelet generation (Di Buduo et al., 2015). In this system, an endothelial lined channel goes through a highly porous silk sponge containing megakaryocytes. ECs promote the formation of platelets through VCAM dependent interactions. Another approach for endothelial/stromal cell interaction was to develop multiple EC-lined channels penetrating through an MSC loaded collagen gel (Kotha et al., 2018). This way, the endothelium and the stromal cells are not separated by a porous membrane, increasing the possibilities for heterotypic interactions. In this study, the authors examined homing behavior of healthy and malignant hematopoietic cells. Their first observation was that primary human derived MSC have a better ability to interact with EC channels compared to the MSC cell line HS5. MSCs also transformed into pericyte-like cells that were lining the endothelium. The extravasation of monocyte was possible in this chip, but uniquely in the presence of stromal cells. Additionally, monocytes were seen cuddling with MSCs demonstrating the close contact interaction between the two cell types. In the same way as with monocytes, HSC and leukemic cell homing was dependent on the niche composition of stromal cells. Influence of the niche composition on cells after extravasation was not investigated in this study.

Kotha et al. (2018) also highlighted a change in EC shape and alignment in presence of different stromal cells, as well as a change in expression of adhesion proteins. Unfortunately, these EC-lined chips did not encourage nor could support gain of insight to the role of vascular organization in the BM. The use of different chips supporting self-organized networks would help to gain insights for vascular organization, by measuring the change in EC adhesion/contraction, vascular length, diameter, branching, barrier permeability and etc. Additional advantage of self-organized vasculature in BM engineering, is the possibility to generate capillary sized vessels, a size more physiologically relevant than the vessels of ∼150 μm diameter generated in EC lined channels (Zheng et al., 2012). Finally, self-organized blood vessels would occupy a 3-dimensional space contrary to preconstructed vessels that are generally planar, and partially lined with glass.

#### Self-Assembled Vasculature

Self-assembled channels in a chip or “vascularized” chips have been widely used and described in the field of vascular biology, as they allow the formation of a 3-dimensional microvascular structures (see vascularization chips, Box 1). Parenthetically, vascularization is almost always generated in bioactive hydrogels (i.e., fibrin, collagen) However the effect of different factor on vascularization can be assessed through the functionalization of chemical hydrogels, with for example VEGF grafted polyethylene glycol (PEG) (García et al., 2016).

**Box 1. Self-assembly in microfluidic chips.**

In tissue engineering, vascularization was found to be a key feature as it is essential for tissue specialization in addition to nutrient and oxygen delivery (Takebe et al., 2015). For proper tissual health and development, the vasculature needs to form a dense network with a mesh size smaller than the diffusion limit of nutrients. This implies that the network must originate from a large channel and then spread out in three dimensions, forming a perfusable tree of small capillaries. Unfortunately, widely used molding technologies have principally allowed the formation of only large flat networks (Takei et al., 2016). To overcome these limitations, microfluidic chips have been developed to promote the self-organization of ECs in a vascular tree structure mimicking physiological organization.

Large progress has been made since, with notably the establishment of supporting conditions such has the structural environment (scaffold or ECM composition), co-cultured cells and interstitial/luminal flow (Kim et al., 2013). Indeed, ECs require specific conditions to form perfusable tubes, and simply aligning EC in the shape of a tree only leads to the formation of a cell chord devoid of lumen (Wu and Ringeisen, 2010). It was shown that the introduction of a structural support in form of a scaffold (Unger et al., 2007) or an ECM (Kim et al., 2013) supports the spontaneous organization of ECs in a perfusable vessel-like structures. Also, the presence of feeder cells supplementing proangiogenic factors in the media such as fibroblasts (Chen et al., 2009) or BM-derived MSCs (Au et al., 2008) also support the formation of vessel tubes. If cultured together, the presence of supporting cells can also bring stability to the formed vessels, notably by lining them in a pericyte like manner (Chamberlain et al., 2012). The vessels that were often formed in an unstable manner, in time were found to be stabilized by the introduction of luminal or interstitial flow into the system (Kim et al., 2013). Indeed, vasculature is extremely responsive to pressure forces like transmural pressure (Wong et al., 2013) that can bring stability, and new vessel sprouts are also controlled through mechanical fluid forces (Song and Munn, 2011). Resuming all these parameters have permitted the reproducible production of a perfusable vascular network in a microfluidic chip. Ever since, these chips have been reproduced in various ways and designed according to the needs (Jeon et al., 2014; Wang et al., 2015; Chen et al., 2017; Oh et al., 2017; Phan et al., 2017; Palikuqi et al., 2020), and have been used for BM engineering (Bersini et al., 2014; Jeon et al., 2015; Jusoh et al., 2015; Nelson et al., 2019; Bessy et al., 2020; Chou et al., 2020; Ma et al., 2020).

These chips have inspired the first BM on a chip models with spontaneous vascularization (Kim et al., 2013; Jeon et al., 2014; Jusoh et al., 2015). In the study by Jeon et al. (2014), the chip was constituted of a central microfluidic channel containing BM-MSC, osteo-differentiated MSCs, and ECs. The channel is lined by pillars that allow a porous separation with the side channels that brings the medium through the formed vasculature and takes it out. A differential pressure between the two side channels also allows the perfusion of the formed vasculature. Therefore, this kind of chip allows a close interaction between endothelial and stromal cells, as no membrane separates the two compartments. Indeed, MSCs were observed in close association with ECs, and with a phenotypic adaptation toward a mural cell lineage, expressing SMC actin. EC change of organization was also recorded, with notably an increased vessel permeability in presence of osteoblastic cells. The perfusion of the vessels, induced EC elongation and actin stress fiber alignment, as well as a reduction of vessel permeability as observed in other vascularized chips. In the study by Jusoh et al. (2015), which included a multiple channel system, ECs were loaded in one of the side channels while the central one was loaded with a mix of fibrin and hydroxyapatite (a mineral form of calcium apatite) to simulate a bone-like environment. Of interest, angiogenesis was observed to be dependent upon hydroxyapatite concentration.

Recent advances in the field have led to the fabrication of complex compartmented chips based on the same principle, achieving the aim to mimic physiological conditions. First, Nelson added a layer of osteoblast to the EC and MSC network, thus generating a new compartment, mimicking a marrow-bone interaction (Nelson et al., 2019). Once again, the overall change of organization of the vascular network, when formed in presence of different supporting cell types, was reported, with notably a vessel thinning to a capillary like structures when in presence of osteoblasts. Another design of engineered BM included additional number of channels to allow the compartmentalization between the bone and vascular regions (Bessy et al., 2020). Glaser et al. (2020) used the supporting property of interstitial flow to promote vasculogenesis in their system. In this chip, three compartments where created, including a vascularized endosteum and marrow compartments with either osteoblasts or stromal cells (Glaser et al., 2020). The two compartments were interacting through diffusion only when mechanical flow forces were not able to pass through the artificial separation and the compartments were equilibrated under pressure. This chip system did manage to generate a vascularized network although it was physically separated from the endosteal and marrow compartments. Ma et al. (2020) also created a multicompartmental chip with a sinus-like mimicking region containing only ECs, a marrow region with malignant hematopoietic B-ALL cells, MSCs and ECs, and an endosteal region with MSCs and osteoblasts. These three regions were organized in concentric circles to mimic long bones marrow organization. However, vasculature assembly, in this chip, displayed a network formation without luminal/tubular formation which doesn’t completely reticulates perfused vascular physiological 3D structure.

Other studies have used the spontaneous self-organization ability of ECs in regular 3D cultures, together with BM stromal cells (Bray et al., 2017). This allowed the reproduction of a complex architectural organization. Yet, in the absence of vascularization guiding channels, this method also failed to achieve successful vessel perfusion. In their study, Marturano-Kruik et al. (2018) have promoted the formation of a vascular network around a decellularized porous calf bone chip, generating a first multimaterial vascular and endosteal niche. The perfusion of this chip during the vascularization process allowed vessel assembly through the entire bone region. Capillary-like structures surrounded by pericyte were observed in a very close proximity to the endosteal surface (within 20 μm). This indicated for the reproduction of a multicellular and multi-material microenvironment susceptible of forming well-defined niches for hematopoiesis. The vascular network organization in this system was thoroughly quantified, in presence and absence of interstitial flow, according to multiple criteria: vessel density, number of junctions, branching index, total vessel length, average vessel length, and the number of endpoints. The interstitial flow supported vasculogenesis with an increase in vessel density and length, increase in the number of junctions, and increased branching index. MSCs organization was observed along vascular structures, with a pericytic specialization markers (presence of NG2+, PDGFRb, and CD146 expressing cells). However, again, the vascular network was not perfusable (Marturano-Kruik et al., 2018).

Overall, these vascularized engineered BM chips have been all used to study various biological aspects under a semi-physiological context. Most of the finding in these studies have been found to be impacted by the overall cellular organization and compound composition in these chips.

One of the key utilizations of these type of chips is the study of cancer cell’s homing, development and resistance to drug and irradiation treatments. Indeed, perfusable vessels can be loaded with cancer cells and their invasion can be recorded. Interestingly, breast cancer cells extravasation do not always correlates with the change in vascular permeability status, which occurs under different culture conditions (Jeon et al., 2015). However, it is quite clear that the local cellular environment strongly impacts breast cancer cell extravasation (Jeon et al., 2015; Glaser et al., 2020). Chips with non-perfused channels can also be loaded with cancer cells to look at the organization of the tumor cells in their niches. Both AML (Bray et al., 2017), B-ALL (Ma et al., 2020) and breast cancer cells (Jeon et al., 2015) were found to be tightly associated with formed vascular blood vessels. The presence of this multicellular organization itself seems to induce or provide cancer cells with the resistance to drug treatment (Bray et al., 2017; Ma et al., 2020) as well as the presence of interstitial flow inducing mechanical signaling (Marturano-Kruik et al., 2018). Bray et al. (2017) also highlighted that the dissociation of tumor cells from vessel, induced by the CXCR4 antagonist AMD3100, can lower the drug resistance effect, generated by the microenvironment. Contact dependent interactions with niche cells is a possible mechanism for tumorogenic drug resistance as well as a possible therapeutic target for malignant niche neutralization in combination with standardized drugs (Ma et al., 2020).

Engineered BM on a chip have also been used to study and reproduce normal hematopoiesis. HSCs homing for instance, have been shown to have different patterning between vascular and endosteal environments (Bessy et al., 2020; Glaser et al., 2020). And additionally, these different environments also differentially regulated hematopoiesis (Glaser et al., 2020). These BM mimicking environments enabled to highlight and decipher a new form of interaction between HSCs and cells from both the endosteal and vascular compartments (Bessy et al., 2020). Finally, these chips were also used to demonstrate stem cell expansion capacity, to measure the impact of the different environments on cellular regulatory processes, to report the effect of distinct environments and different types of drugs over HSC mobilization, and to investigate the radioprotective effects of different niche cells (Nelson et al., 2019; Glaser et al., 2020).

A significant breakthrough was recently achieved in a study by Palikuqi et al. (2020), in which the authors generated self-assembling, tissue adaptable, durable, and perfusable human vascular capillary network, named ‘Organ-On-VascularNet.’ It is common knowledge that *ex vivo* cultured ECs lose their heterotypic tissue specificity and some vascular capacities (such as *in vitro* tubular network formation). In this study, the authors introduced the ETS variant transcription factor 2 (ETV2) into adult ECs. Overexpression of ETV2 in *ex vivo* cultured ECs resets them into an “embryonic reversed” state (reset vascular ECs, R-VEC), resembling embryonic ECs primed for further tissue adaptation and specification. In addition, through transcriptional activity and chromatin modification, ETV2 activates tubulogenic programs (e.g., RAP1), which in turn promotes the self-assembly of lumenized and durable capillary structures. This reprogramming approach driving vascular ECs just one step backward, while avoiding re-entry and transition through a pluripotent state, allows vascular niche heterotypic re-specification to accommodate and functionally support a variety of organoids and tumoroids. Although the system has been demonstrated in the context of multicellular solid organoids and tumors, it is tempting to assume that with the right cellular and chemical composition it would recapitulate the BM vascularized environment. Moreover, the transcriptional signature of ETV2-induced R-VECs exhibits characteristics of arterial-capillary type of ECs (from which other types of ECs develop during embryogenesis) allowing introduction of pericytic-like MSCs (another pivotal HSC niche component) into the system along with HSCs. However, as distinct types of vascular niches were shown to dictate different developmental fate choice decisions and metabolic status of HSCs (Itkin et al., 2016), with the adequate “reprogramming” tools R-VEC may potentially be directed into venular or further even into lymphatic fates, allowing more extensive interrogation of distinct sub-types of niches in a defined organ. Of course, further heterotypic single cell analysis of different endothelial sub-types in various organs will help to define tissue and sub-type specific transcription factors that can serve as directive tools for engineering of tissue and function specialized niches.

The self-assembly system has the advantage of generating perfusable capillary like structures with very high similarity to physiological structures, when compared to other microfabrication methods. However, this self-assembly based system highlights the fact that EC organization is highly variable and dependent on the culture conditions in addition to other parameters, which makes it challenging to compare analysis results from different studies. The introduction of “engineered border” or standardizing this system may allow the generation of a reproducible vascular network that has the normalization required for an extended comparison between two different conditions. The normalization of a system through “border” control is a solution that has been previously reported in other self-assembled systems such as organoids (Brassard and Lutolf, 2019). However, capillaries are on a different size scale compared to organoids, and the vascular structures produced by classical engineering methods are an order of magnitude larger than physiological structures (channels size is ∼150–200 μm). Recent advances in bioprinting could be a solution to engineer thin spatially organized microstructures. In addition, printing allows a non-random distribution of cells which could lead to the generation of models with a higher degree of organization.

#### Bioprinting

Bioprinting studies are usually focused toward regenerative medicine goals and thus on the generation of large size samples, however, it has also been used for the generation of small organ-on-a-chip models. The great advantage of this approach is the possibility to engineer thin spatially organized microstructures with a non-random cell distribution, generating models with a high degree of organization. Surprisingly, bioprinting technology has not been applied yet to engineer a BM chip model, even though its multiple materials approach could be the solution to the complexity of the BM microenvironment.

The endosteum is a crucial part of the BM and is often distinguished only based on the presence of osteo-differentiated MSCs. One option to palliate this missing element, from BM vascular chips, is to introduce severed and decellularized bones (Marturano-Kruik et al., 2018). Another option would be to introduce a controlled deposition of bone elements by bioprinting. Various options are available to print bone-like material, for example printing of ECM proteins coated with hydroxyapatite, or by printing directly hydroxyapatite together with a supporting biodegradable polymer like polycaprolactone (PCL) or polylactic-co-glycolic acid (PLGA). In both cases, scaffold structures can be printed and seeded with MSCs, that will differentiate toward osteoblastic cells in culture (a process supported by the presence of hydroxyapatite) (Yao et al., 2015; Jakus et al., 2016; Luo et al., 2018). Scaffold polymers can also be seeded with osteoblastic cells to allow calcium deposition and ECM proteins that will in turn support the functionalization of the vascular structure. All in all, bone printing has been elucidated as it does not require a particularly small resolution. However, the physical properties of the material and the conditions necessary for printing (either heat or in presence of solvents) challenges further development of direct cell printing on a chip.

As expected, the greater challenge comes in the printing of a vascular system of the scale of capillaries. Once again, most vascular bioprinting studies focus on the reproduction of large channels for regenerative medicine. The generation of capillary sized vessels is not possible by direct extrusion printing as it would require the deposition of single cells in a very precise location, which implies using a thin needle generating large amounts of shear stress over the cells. The only possibility for direct printing is the laser assisted cell deposition, which allows the alignment of single cells than can then self-assemble in a tubular structure that will follow a guiding printed pattern (Guillotin et al., 2010; Wu and Ringeisen, 2010). Yet, extrusion bioprinting is a more affordable option, and it is also more versatile as it allows multiple materials printing, a necessary function for BM engineering. With extrusion printing, the use of an indirect approach is necessary, for example the use of sacrificial materials. It has the advantage of allowing the formation of tubes of the same diameter of that of the printed filament. Subsequent seeding of ECs further leads to the formation of lumenized vascular tubes. The use of both Pluronic-F127 (Wu et al., 2011) and carbohydrate glass (Miller et al., 2012) as sacrificial material have allowed the generation of hollow lumens of various diameters with a resolution limit of approximately 20 μm. However, no picture of these small channels coated with ECs has been presented yet.

Another key advantage of bioprinting a BM chip, is the possibility to generate BM specialized types and sub-types of vessels. Indeed, engineered BM have so far only focused in introducing a perfusable vascular network, but have not distinguished the subvascular entities despite the numerous demonstration that arterioles and sinusoids, for example, play different roles in regulation of hematopoiesis (Kunisaki et al., 2013; Acar et al., 2015; Itkin et al., 2016). One important difference between these vascular structures resides in the supporting cells, surrounding the endothelial layer, inducing a change in vascular organization, leading to formation of vessels of different size, permeability, function, and production of specific angiocrine factors to support distinct hematopoietic fates. Conveniently, bioprinting allows multilayer deposition allowing the formation of tubes coated with a layer of various cell types (SMCs, MSCs, fibroblasts, etc.) (Kolesky et al., 2014; Gao et al., 2017; Schöneberg et al., 2018). The deposition of a layer of stromal cells may lead to a homogeneous coating of the vascular tube in predefined regions, therefore creating different vascular structures along the same tubular vessel.

The main interest of bioprinting still resides in the possibility of designing chips with multiple types of materials harboring different physical properties. However, only few studies aimed at reproducing a vasculature within an endosteal environment, and none of these studies have actually aimed at reproducing a BM tissue. In their model for vascularized bone generation, Cui et al. (2016) proposed a dual printing of a rigid polymer and a soft cell-laden hydrogel. The polymer was loaded with BMP-2 to promote later stage ossification. The chip was designed with open macrostructures allowing the annular printing of an EC containing hydrogel. The obtained structure was kept in culture in presence of an interstitial flow which supported the formation of vessel capillaries. However, this fully vascularized bone structure could not be perfused and the capillaries were not formed as the result of vasculogenesis which could not be controlled spatially. Other studies solely printed a bone scaffold and seeded ECs to generate a capillary network. Under these conditions, even if there is no control of the vascular tree outgrowth, the bone scaffold guided angiogenesis patterning (Chiesa et al., 2020). In other studies some effort was made in the bone scaffold design to promote and guide neovascularization (Zhang et al., 2017).

**FIGURE 4:**
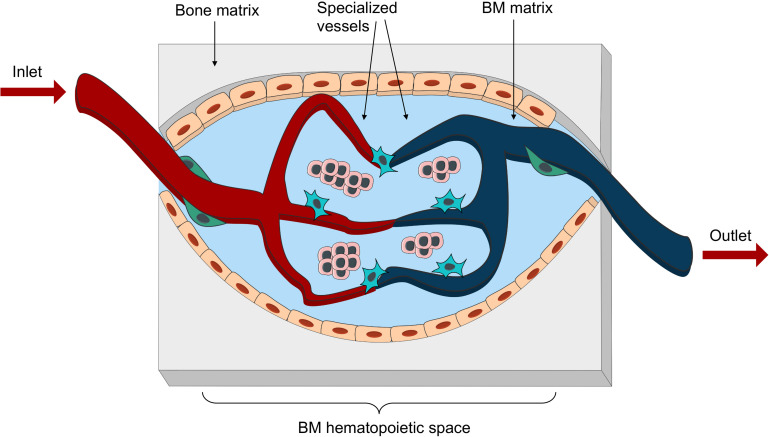
Bioengineered vascularized BM niche unit. Schematic representation of the essential elements to be reproduced in an engineered vascularized BM niche-on-a-chip. These would include different matrix for bone and marrow space, as well as an intricate net of specialized vessels in close proximity with stromal and hematopoietic components.

In summary, these *in vitro* promising studies demonstrate thehigh versatility of biological engineering tools, especially in theera of multi material printing and cellular reprogramming, for thegeneration of *ex vivo* organized tissues on a chip (Tables 2, 3). The next avenue would be to use these relatively new technologies for the generation of a vascularized BM-on-a-chip unit which could closely model the human BM with its full cellular and extracellular organization (Figure 4).

**TABLE 2:** Supporting materials.

Material	Application	Technology	Manufacturing complexity	Commentaries	References
Fibrin	*in vitro*	Microfluidic chip	++	Generation of interconnected compartments with different vasculature-associated stromal cells No focus on vascularization MSCs differentiate in mural cell lining the vasculature	Ma et al. (2020) de la Puente et al. (2015) Jeon et al. (2015)

Fibrin / Hydroxy apatite	*in vitro*	Microfluidic chip	++	Hydroxyapatite increases angiogenesis *in vitro*	Jusoh et al. (2015)

Collagen I	*in vitro*	Inverted vessels Microfluidic chip	++	Flow causes a transient endothelial cell activation, perhaps because of its non-laminar nature. Endothelial cells elongation in the direction of the flow	Khan et al. (2012) Kotha et al. (2018)

Alginate Matrigel Gelatin MA	*in vitro*	3D hydrogels	+	Matrigel supported cell-cell interaction leading to a better network formation	Braham et al. (2019)

Hyaluronic acid/Gelatin	*in vitro*	Hydrogel in a microfluidic chip	+++	Generation of inverted vessels with arteriolar and sinusoidal endothelial cells	Aleman et al. (2019)

Collagen I/Matrigel	*in vitro*	Microfluidic chip	++	CXCR2 dependent extravasation of cancer cells through the vasculature	Bersini et al. (2014)

Fibrin / collagen I	*in vitro*	Microfluidic chip	++	Hematopoietic cells egress through endothelial cell coated channels Characterization of the vascular network organization amelioration in presence of both OB and MSCs Detailed observation of HSPC/endothelial cell interactions	Chou et al. (2020) Nelson et al. (2019) Bessy et al. (2020)

Silk / Collagen IV / Laminin / fibronectin	*in vitro*	3D hydrogel with a channel	++	Observation of various Hematopoietic cell interaction with vasculature	Di Buduo et al. (2015)

StarPEG-heparin	*in vitro*	Commercial hydrogel	+	Hydrogel can easily be functionnalized with RGD peptides for cell remodeling through MMPs	Bray et al. (2017)

Decellularized bone matrix	*in vitro*	Microfluidic chip	++	Angiogenesis of sinusoid-like vessels in close vicinity of the bone	Marturano-Kruik et al. (2018)

Polylactic Acid / Matrigel	*in vitro*	Bioprinting / SLA	+++	Guided angiogenesis in a hard scaffold	Cui et al. (2016)

Gelatin nanohydroxyapatite Gelatin MA/fibrin	*in vitro*	Bioprinting	+++	Bone scaffold organisation directs angiogenesis	Chiesa et al. (2020)

Matrigel embedded starch–poly (caprolactone) scaffold	*in vivo*	Sub-cutaneous implantation in mice	++	Good vessel formation anastomosed with host vasculature	Ghanaati et al. (2010)

fibronectin-collagen I scaffold	*in vivo*	Sub-cutaneous implantation in mice	+	Good vessel formation but lack of bone structure/matrix	Koike et al. (2004), Au et al. (2008)

Matrigel	*in vivo*	Sub-cutaneous implantation in mice	+	Good bone structure/matrix, lack of human vasculature formation	Chen et al. (2012), Reinisch et al. (2016, 2017)

Alginate-MA / Star PEG-MA	*in vivo*	Sub-cutaneous implantation in mice	++	Good bone structure/matrix, lack of human vasculature formation	Shah et al. (2019)

Collagen sponge	*in vivo*	Sub-cutaneous implantation in mice	+	Good cartilage formation, lack of human vasculature formation	Fritsch et al. (2018), Bourgine et al. (2019)

Gelatin sponges	*in vivo*	Sub-cutaneous implantation in mice	+	Good bone structure/matrix, human vasculature anastomosed to host murine one	Abarrategi et al. (2017), Passaro et al. (2017a)

Hyperelastic bone (Hydroxyapatite/PLGA or PCL)	*in vivo*	Bioprinting and sub-cutaneous implantation in mice	+++	Good cartilage formation, good anastomosis in presence of MSCs	Jakus et al. (2016), Nulty et al. (2021)

3D printed scaffolds coated with star-PEG hydrogels	*in vivo*	Orthotopic BM implantation in mice	+++	Regressed human vasculature, overtaken by murine one	Baldwin et al. (2017)

beta-TCP, BRT Bioceramic	*in vivo* / *in vitro*	Bioprinting and sub-cutaneous implantation in mice / orthotopic implantation in rabbits	+++	Scaffolds guides angiogenesis, *in vitro* and *in vivo*	Zhang et al. (2017)

Laminin, entactin, collagen IV	*in vivo* / *in vitro*	Sub-cutaneous implantation in mice/Microfluidic chip	++	Functional human vasculature anastomized with murine one	Palikuqi et al. (2020)

Demineralized bone powder, collagen I	*in vivo*/ *in vitro*	Sub-cutaneous implantation in mice / microfluidic chip	++	Good bone structure/matrix, lack of human vasculature and follow up on vascular functionality	Torisawa et al. (2014)

**TABLE 3:** Bioengineering approaches.

Technology	Materials	Domain	Application	Pros	Cons	References
3D hydrogel with a channel	Silk / Collagen IV / Laminin / fibronectin	*in vitro*	Interactions between hematopoietic cells and niche cells, extravasation into the circulating media	Presence of a barrier creating sites for intercompartment interactions	No presence of cellularized endothelial tubes	Di Buduo et al. (2015)

3D hydrogels	Alginate, Matrigel, Gelatin MA, StarPEG-heparin	*in vitro*	Heterotypic cell interactions, biochemical mechanisms	Very simple to implement, multiple interactions assays are possible	Vessels are not anastomosed to a feeding source	Bray et al. (2017), Braham et al. (2019)

Bioprinted / SLA bone matrix with self assembled vessels	Gelatin nanohydroxyapatite Gelatin MA/fibrin Polylactic Acid / Matrigel	*in vitro*	Fabrication of vascularized matrices within ossified material, can be used for grafts and cancer development	Possible generation of pseudo-organs with controlled geometry of the different compartments and guided vasculature	Perfusion system isn’t connected to the vasculature Direct printing hasn’t achieved the resolution necessary for the formation of a capillary vascular network.	Chiesa et al. (2020) Cui et al. (2016)

Bioprinting and sub-cutaneous/orthotopic implantation in mice/rabbit	Hyperelastic bone (Hydroxyapatite/PLGA or PCL) beta-TCP, BRT Bioceramic	*in vivo*/ *in vitro*	Bone environment reconstitution *in vivo*	Reconstruction of anastomosed organ for possible engraftment	The absence of perfusion system prevents *in vitro* investigations. Direct printing hasn’t achieved the resolution necessary for the formation of a capillary vascular network.	Jakus et al. (2016) Zhang et al. (2017)

Microfluidic chip with inverted vessels	Hyaluronic acid/gelatin, collagen I	*in vitro*	Cancer cells invasion / intravasation	Simple set up, possibility to compare different sources of endothelial cells	No tubular formation of endothelial cells, organisation investigations are impossible	Aleman et al. (2019), Khan et al. (2012)

Microfluidic chip with patterned vasculature	Collagen I	*in vitro*	Niche cells interaction, hematopoietic and leukemic cell extravasation	Controlled vascular organisation, assay standardization	Resolution limit in vessel size, non physiological organisation of endothelial cells	Kotha et al. (2018)

Microfluidic chip with self-assembled vasculature	Collagen I, fibrin, fibrin / hydroxyapatite, Matrigel, decellularized bone matrix	*in vitro*	Hematopoietic cells interaction with niche cells Vasculature dynamics, organisation, permeability… Cancer cell invasion, extravasation and response to drugs Niche response to radiotherapy	Highly versatile devices, moderately easy to establish in a laboratory. Possibility of fully perfused vascular system.	Few studies have included an endosteal compartment. Self-assembled vessels are not guided to follow native bone marrow organisation. Chip content cannot be retrieved easily and has not yet been used as a graft.	Bersini et al. (2014), Jeon et al. (2015), Jusoh et al. (2015), de la Puente et al. (2015), Ma et al. (2020), Marturano-Kruik et al. (2018), Palikuqi et al. (2020)

Bioprinted BM orthotopic implantation in mice	Star-PEG hydrogel, polycaprolactone	*in vivo*	Only example of full bone marrow graft	Possibility to control geometry and organisation of the graft. The use of various materials brings the appropriate mechanical properties.	Regressed human vasculature, overtaken by murine one. Difficult to implement, use of non-biological materials	Baldwin et al. (2017)

Sub-cutaneous scaffold implantation in immunodeficient mice	Matrigel embedded starch–poly (caprolactone) scaffold, fibronectin-collagen I scaffold, Matrigel, collagen sponge, gelatin sponge, laminin – entactin – collagen IV matrix	*in vivo*	Hematopoietic stem cell grafts and homing Vasculature organisation, and anastomosis with host vasculature Hematopoietic stem cell production	Highly versatile system. Possibility to include different growth factors to direct cell differentiation. Possibility to do long terms assays.	Heterotopic site of implantation. Little control on vascular formation. Long setting up time. Chimeric human-mouse vasculature	Koike et al. (2004), Au et al. (2008), Ghanaati et al. (2010), Chen et al. (2012), Reinisch et al. (2016, 2017), Abarrategi et al. (2017), Passaro et al. (2017a), Fritsch et al. (2018), Bourgine et al. (2019), Shah et al. (2019)

Sub-cutaneous implantation in mice / microfluidic chip	Demineralized bone powder, collagen I	*in vivo*/ *in vitro*	Only example of engineered BM alternatively grafted and studied in a microfluidic device	Having the graft in a microfluidic system allow follow-up experiments impossible in other devices.	The vascular system is not connected to the perfusion system.	Torisawa et al. (2014)

### In vivo

As for *in vivo* studies, the main aim of bioengineering an ectopic BM niche has been to support human hematopoiesis for either experimental systems allowing long term studies focusing on HSC-niche interaction, or to achieve the unmet need of optimized *ex vivo* platform for HSC robust expansion while maintaining stemness and multipotent developmental potential. The endpoint for these studies is usually at the time point when stemness gives in to differentiation, which can be held back for limited time frame by the presence of a minimal BM-like tissue components obtained from the combination of human MSCs and diverse biomaterials (as reviewed in Abarrategi et al., 2018). In most of these cases, very little experimental effort has been done to properly characterize and improve the vascularization of these systems. Nevertheless, once some of these experimental systems were further transplanted *in vivo* into immunocompromised mice, the presence of an ingrown murine vasculature derived from either the migration of circulating EC into the grafts or neoangiogenesis of neighboring blood vessels, has been described as an essential component for the survival and prosperity of these tissue grafts. Otherwise these grafts would remain devoid of nutrients and oxygen. Recent advances have largely expanded the current knowledge of the BM vasculature, which is now widely recognized as an essential component of a functional homeostatic BM, providing the required support for the maturation and steady state maintenance of the tissue, directing the correct differentiation pattern of BM residing MSCs and HSCs as well as the production of matrix, cytokines and growth factors (Langen et al., 2017; Chen et al., 2019). All these properties are likely to impact on the ability of ectopic niches to support human HSC graft’s homing, quiescence and differentiation (Itkin et al., 2016).

Successful production of human vasculature in a graft was achieved by using mesenchymal and endothelial cells derived from the stromal vascular fraction of human adipose tissue (Ghanaati et al., 2010). The authors have applied a Matrigel embedded starch–poly(caprolactone) scaffold to create vascularized *in vivo* structures anastomosed with the murine vasculature as soon as 2 days after implantation. However, the vessels in these grafts seemed to achieve full connectivity to the host vasculature only 2 weeks later.

Similarly, Koike et al. (2004) have used HUVEC in association with a mesenchymal precursor cell line to produce human vascularized tissues in fibronectin-collagen I scaffolds and have shown that only after 4 weeks post implantation the vessels that were assembled in these grafts became functional. The same group achieved a similar result by applying human BM-derived MSC as a source of perivascular cells (Au et al., 2008). However, these constructs lacked the generation of a bone component, together with all the environmental impact on BM vascular specification.

Reinisch et al. (2016, 2017) generated BM-like ossicles using Matrigel as supportive material (). This system has the advantage of easy implantation in mice with a minimally invasive procedure. Moreover, the supporting scaffold is a standardized and has a relatively wide-ranging material. Using a similar system, Chen et al. (2012) have introduced peripheral blood circulating ECs (PB-ECs) into the scaffold to reconstitute a humanized vascular network, which was proven to be functional for delivery of probes as well as human cells. However, the downside of this model is the necessity to irradiate the animals prior to hematopoietic cell implantation, a process favoring human engraftment but also leading to a dramatic disruption of endogenous BM vascular structure and function. Although mimicking BM vasculature was not amongst the aims of Reinisch study, the authors mention that scaffold human vascularization was supported by the murine host system during 2 weeks post implantation through the arrival and homing of BM-MSCs, as assumed from the increase in pro-angiogenic factors derived from MSCs (Reinisch et al., 2015). However, no vascular formation was observed in scaffolds seeded with MSC derived from cord, skin or adipose tissue. Given that all the previously mentioned reports proved that different sources of MSCs can functionally lead to human vascular network formation, one could speculate that this ability is directly linked to the response of human ECs to a specific BM-derived type of MSC, rather than an intrinsic property of MSCs.

Another injectable BM niche graft model has been recently reported (Shah et al., 2019), where the authors have implemented an alginate-PEG scaffold incorporating specific ligands to support T cell development and bone formation. Also, in this study, an ingrowth of murine vasculature was induced, yet no detailed characterization of its vascular structure was performed.

Adaptable sponges are another an intriguing option for *in vivo* BM tissue generation, with a reticular network of collagen I (Fritsch et al., 2018; Bourgine et al., 2019) or with gelatin (Abarrategi et al., 2017). These materials were suggested to “mature” into BM-like matrices *in vivo* or *in vitro* via MSCs remodeling, while the contribution of ECs to this “maturation” process was not well defined. The collagen-based scaffold used by Bourgine et al. (2019) developed in human cartilage *in vitro* upon culture with modified BM-derived human MSC expressing SDF-1 (CXCL12). The absence of ECs likely impacts on tissue maturation. Once implanted *in vivo*, these scaffolds became highly vascularized by infiltrating ECs, as shown by confocal microscopy (Bourgine et al., 2019). Abarrategi et al. (2017) used a surgery-based implantation technique to transplant *in vivo* a gelatin scaffold coated with human BM-derived MSCs in the presence or absence of osteo-inductive BMP-1. While in their first report the authors assessed the hematopoietic support potential by the BM-like grafts (Abarrategi et al., 2017), in the following up protocol study (Passaro et al., 2017a) additional human EC component (E4ORF1 HUVEC, Seandel et al., 2008) was incorporated. Functional perfused human vessels, anastomosed with murine vasculature, were obtained using this method, associated with human hematopoietic cells and MSCs. However, structural characterization of these vessels as well as a thorough description of their heterogeneity has yet to be performed.

In between *in vivo* and *in vitro* approaches, Torisawa et al. (2014) fabricated a ‘bone marrow–on–a–chip’ tissue derived from *in vivo* implanted PDMS scaffolds, then explanted and perfused in a microfluidic device. While this approach has the key advantage of generating a geometrically defined tissue *in vivo*, highly resembling the BM, its subsequential *in vitro* part has not been characterized in terms of vascular reperfusion, which is a major challenge in growing tissue explants (Torisawa et al., 2014).

In an attempt to reproduce the complex periosteal BM architecture, Baldwin et al. (2017) designed a multi-material platform combining two communicating cellular components representing the periosteal osteoprogenitor and the vascular niche. The choice of orthotopic implantation is one of the great implementations of this method, which together with the use of a 3D printed scaffolds, coated with star-PEG hydrogels, allowed a physiological-like incorporation of the cells and the remodeling of the grafts, *in vivo*. However, the *in vitro* generation of a human vascular networks prior to implantation is surprisingly counterproductive, since human ECs in this system have lost their proliferative and expansion capacity and thus were taken over and outcompeted *in vivo* by the host murine vasculature. Another explanation of this gradual regression of human vessels could be the fact that they were physically separated from MSCs in this study, impairing the physiological perivascular support necessary to sustain angiogenesis and vascular formation.

To overcome this issue, Nulty et al. (2021) have recently combined a 3D printed polycaprolactone (PCL) scaffolds with human MSCs and HUVEC embedded in a fibrin hydrogel. These devices were next implanted *in vivo* in critically sized femoral bone defects in rats. Authors have observed that only bioprinted tissues containing both HUVECs and hBMSCs, that were first allowed to mature *in vitro*, supported robust blood vessel development *in vivo* (Nulty et al., 2021).

Successful *in vivo* implantation was also achieved with the R-VEC system described above (Palikuqi et al., 2020). When R-VECs were embedded in predefined matrix (composed of laminin, entactin, and collagen IV) and were transplanted *in vivo*, they anastomosed with murine vasculature, acquired a supportive layer of murine-derived pericytes, and established a patterned and durable, months lasting vasculature (Palikuqi et al., 2020). In the same study the authors have used R-VECs to repopulate decellularized rat intestinal scaffolds. After 1 week of *ex vivo* culture, allowing scaffold vascularization, these scaffolds were further transplanted *in vivo*. R-VEC vascularized scaffolds anastomosed to murine vasculature and exhibited durable arborization of engrafted tissue scaffolds. Thus this system holds a promising potential for vascularization of decellularized human bones, an approach that has so far only been focusing on the human mesenchymal component (Nakamura et al., 2019).

Further development of 3D printing techniques was performed by Cidonio et al. (2020), who used nanoclay-based bioink in association with alginate and BMP-2 to recreate an environment stimulating the osteogenic differentiation of BM-MSCs. The authors were able to reproduce a BM-like tissue *in vivo* upon subcutaneous implantation. However functional investigation of 3D printed scaffold vascularization was performed only in an *ex vivo* chorioallantoic model. Similarly, Jakus et al. (2016) have developed a hyperelastic bone, which was constituted mostly by hydroxyapatite, naturally present in human bones. Human MSCs in contact with this scaffold material further differentiated toward osteolineage cell progeny, and further *in vivo* implantation of these tissue scaffolds allowed ingrowth of functional vasculature (Jakus et al., 2016).

In summary, although very little has been achieved *in vivo* in terms of human vascularization, the great advances in the field of biomaterials and the extensive knowledge recently acquired in BM vascular biology foresee the advent of a novel generation of bioengineered tissue recapitulating a *bona fide* BM vasculature (Tables 2, 3).

## Clinical Applications and Future Perspectives

As of today, most clinical trials in the context of endothelium apply pro- or anti-angiogenic strategies, manipulating angiogenic signaling pathways to promote vascular recovery or to inhibit the development of tumorigenic vasculature (Carmeliet and Jain, 2011). Yet, for hematological malignancies treatment, myeloablative therapies such as chemotherapy and irradiation are applied, resulting with the disruption of the BM vascular architecture and the BM niche (Hooper et al., 2009). As it was shown in murine models that transplantation of young ECs mitigates HSC aging effects by the aged BM niche ECs (Poulos et al., 2017), it is very tempting to assume that EC transplantation could mitigate irradiation derived vascular insults, not only in the BM but also in other vital organs (Rafii et al., 2016). Recently, an abstract has reported a phase I clinical trial with GMP grade product of E4orf1 HUVEC, transplanted into lymphoma patients undergoing high dose of irradiation therapy followed by autologous HSPC transplantation. No side effects due to EC transplant were reported, EC transplant improved toxicity parameters and reduced irradiation-mediated side effects, while augmenting faster neutrophil and platelet engraftment/recovery (Scordo et al., 2020). Thus, cellular EC therapy is becoming a very attractable clinical possibility to reconstitute functional blood vessels, delivering oxygen and nutrients to injured tissue, to re-establish a functional niche entity, providing angiocrine factors for recovering stem cells, and to mitigate irradiation or similar insults resulting with tissue damage such as fibrosis and tissue scaring.

Engineering stable ECs such as E4orf1 ECs with enhanced niche supportive capacity may provide a short-term solution for engrafting HSPCs. For long-term applications, an engraftable line of ECs needs to be designed, such as the R-VEC model with tissue adaptable capabilities and preferably with immunosuppressive properties to exclude immune-mediated graft rejection. Although the most suitable ECs for such purpose would be autologous, adult ECs, especially from elderly individuals, are very hard and challenging to isolate, culture, and expand *ex vivo* for such modification. A recent abstract reports the possibility to establish autologous BM-derived EC cultures from donors and immortalize them for long-terms culture and genetic editing (Schaeffer et al., 2020). Another possibility would be to “reprogram” and convert easily accessible adult MSC or hematopoietic cell populations into EC-like cells with vascular EC functions and vessel-engraftable properties, as was performed experimentally with amniotic-origin derived cells (Ginsberg et al., 2012).

Human vascularized BM-chips replicating HSPC’s microenvironment, with MSC, ECs and mature hematopoietic cells in presence, can be applied for efficient drug screening (as a pre-step or part of a clinical trial) for molecules aiming to mitigate the beneficial and protective interaction between a malignant type of hematopoietic cell to its corrupted niche. In this type of setting researchers won’t have to rely on animal “physiological” models which do not always reproduce the same results once clinical trials move forward to human subjects. For example, new types of small molecule inhibitors for CXCR4 can be tested by perfusion into a BM-chip platform to hamper the adhesion and protection of AML cells in the BM microenvironment, to visualize mobilization of exposed AML blasts into the assembled vessels in these BM-chips, and to examine by multiomics approach how CXCR4 blockade using small molecule inhibitors withdraws molecularly mediated chemoresistance of AML blasts, following chemotherapeutic perfusion of BM-chips (Chen et al., 2013; Borthakur et al., 2020).

Another exciting possibility would be to clinically apply as transplantable graft a multicellular vascularized BM chip. Standard HSPC transplantation protocols dictate infusing the harvested cells, containing the long-term repopulating HSCs, intravenously into previously “primed” patients. “Priming” usually consists of chemotherapeutic and/or irradiation treatments which likely harm blood vessel lining ECs and result with hampered vascular niche function for expanding and differentiating HSPCs, and with enhanced vessel permeability and tissue leakiness. Additionally, intravenously transplanted HSPCs might wedge, during their travels in the circulation, in sites like the small lung capillaries, possibly reducing the yield and numbers of HSPCs that manage to successfully home into the BM and lodge into their niche. Also, prolonged exposure of transplanted HSPCs to blood plasma in circulation due to niche lodgment failure, or in the BM due to higher blood fluid leakiness, can abrogate stem cell function and properties via enhancing reactive oxygen species (ROS) levels in HSPCs (Itkin et al., 2016; Passaro et al., 2017b). Transplanting BM-like grafts, containing the pivotal components of the niche together with expanding HSPCs, will provide temporary “shelter” and protection for HSPCs while performing as a subordinate hematopoietic site, producing blood cells. Upon anastomosis and full perfusion between the patient’s vasculature and the transplanted grafts, HSPCs should be able to eventually safely migrate and repopulate patient’s BM. This “protective” approach, of HSPC transplantation with their temporary essential facilities and a “home” supportive structure, may actually result with the requirement for reduced number of HSCs per transplant by ensuring a better engraftable yield, allowing the use of lower doses per graft, and might even enable the use of a single cord blood dose per adult.

However, cellular EC or BM-chip therapeutic application and clinical studies are now in the initial phase of design, taking first baby footsteps in the field. Thus, potential adverse effects should be considered and included in any future study design and analysis. A potential limitation that needs to be considered is the source of ECs for such cellular therapies and for transplantable BM-chips, as autologous adult EC isolation, culturing, and engineering is challenging when comes to large scale of cell numbers required for human application, especially from elderly patients. This limitation might require the generation of a “universal” HUVEC line, “invisible” to the immune system (HLAs depleted), serving as a global donor for cellular therapies and BM-chip designs, while still allowing amplification and expansion of enough ECs for human therapeutics.

The clinical application of *ex vivo* engineered vascular systems would be the optimal transition from “bench” to “bed-side,” translating basic scientific developments into therapeutical protocols.

## Author Contributions

TB, TI, and DP reviewed the field and wrote the manuscript. All authors contributed to the article and approved the submitted version.

## Conflict of Interest

The authors declare that the research was conducted in the absence of any commercial or financial relationships that could be construed as a potential conflict of interest.
